# *SMAD4* haploinsufficiency in small intestinal neuroendocrine tumors

**DOI:** 10.1186/s12885-021-07786-9

**Published:** 2021-01-28

**Authors:** Tobias Hofving, Erik Elias, Anna Rehammar, Linda Inge, Gülay Altiparmak, Marta Persson, Erik Kristiansson, Martin E. Johansson, Ola Nilsson, Yvonne Arvidsson

**Affiliations:** 1 Sahlgrenska Center for Cancer Research, Department of Laboratory Medicine, Institute of Biomedicine, Sahlgrenska Academy at University of Gothenburg, Box 425, SE-405 30 Gothenburg, Sweden; 2 Department of Surgery, Institute of Clinical Sciences, Sahlgrenska Academy at University of Gothenburg, Gothenburg, Sweden; 3 Department of Mathematical Sciences, Chalmers University of Technology and University of Gothenburg, Gothenburg, Sweden

**Keywords:** SINET, SMAD4, Haploinsufficiency, Chromosome 18, Tissue microarray, FISH

## Abstract

**Background:**

Patients with small intestinal neuroendocrine tumors (SINETs) frequently present with lymph node and liver metastases at the time of diagnosis, but the molecular changes that lead to the progression of these tumors are largely unknown. Sequencing studies have only identified recurrent point mutations at low frequencies with *CDKN1B* being the most common harboring heterozygous mutations in less than 10% of all tumors. Although SINETs are genetically stable tumors with a low frequency of point mutations and indels, they often harbor recurrent hemizygous copy number alterations (CNAs) yet the functional implications of these CNA are unclear.

**Methods:**

Utilizing comparative genomic hybridization (CGH) arrays we analyzed the CNA profile of 131 SINETs from 117 patients. Two tumor suppressor genes and corresponding proteins i.e. *SMAD4,* and *CDKN1B,* were further characterized using a tissue microarray (TMA) with 846 SINETs. Immunohistochemistry (IHC) was used to quantify protein expression in TMA samples and this was correlated with chromosome number evaluated with fluorescent in-situ hybridization (FISH). Intestinal tissue from a Smad4^+/−^ mouse model was used to detect entero-endocrine cell hyperplasia with IHC.

**Results:**

Analyzing the CGH arrays we found loss of chromosome 18q and *SMAD4* in 71% of SINETs and that focal loss of chromosome 12 affecting the *CDKN1B* was present in 9.4% of SINETs. No homozygous loss of chromosome 18 was detected. Hemizygous loss of *SMAD4*, but not *CDKN1B*, significantly correlated with reduced protein levels but hemizygous loss of *SMAD4* did not induce entero-endocrine cell hyperplasia in the Smad4^+/−^ mouse model. In addition, patients with low SMAD4 protein expression in primary tumors more often presented with metastatic disease.

**Conclusions:**

Hemizygous loss of chromosome 18q and the *SMAD4* gene is the most common genetic event in SINETs and our results suggests that this could influence SMAD4 protein expression and spread of metastases. Although *SMAD4* haploinsufficiency alone did not induce tumor initiation, loss of chromosome 18 could represent an evolutionary advantage in SINETs explaining the high prevalence of this aberration. Functional consequences of reduced SMAD4 protein levels could hypothetically be a potential mechanism as to why loss of chromosome 18 appears to be clonally selected in SINETs.

**Supplementary Information:**

The online version contains supplementary material available at 10.1186/s12885-021-07786-9.

## Background

Gene mutations contributing to cancer development and progression are termed driver mutations. For many tumor types specific driver mutations have been determined and validated, thus describing molecular mechanisms for tumorigenesis and posing as potential targets for therapeutic interventions. Small intestinal neuroendocrine tumors (SINETs) originate from enterochromaffin (EC) cells in the small intestinal mucosa and are generally slow-growing neoplasms. Compared to many tumor types, SINETs are genetically stable with a somatic nonsynonymous mutational frequency more than 10-fold lower than that of cutaneous melanomas, squamous cell carcinomas and adenocarcinomas of the lung [1]. The stability of SINETs in terms of point mutations is further illustrated by the fact that only a few genes have been found mutated in more than one patient tumor. Among recurrently mutated genes *CDKN1B* has been reported with the highest frequency, mutated in only about 7.8–9.6% of all SINETs [2, 3]. Consequently, for a large proportion of SINETs, no apparent gene driver mutations have been identified. While recurrent point mutations have only been identified for a few genes, SINETs instead harbor several recurrent hemizygous whole chromosomal CNAs [4–6]. The classical two-hit hypothesis suggests that both alleles of a tumor suppressor should be inactivated to promote cancer development [7]. There are however several prominent examples of tumor suppressor genes that do not follow this rule. Two such genes are *PTEN* [8] and *P53* [9], which have been confirmed to be haploinsufficient, meaning that cells with one inactivated gene copy does not maintain their wild-type phenotype. The most frequently occurring genetic alteration in SINETs is hemizygous loss of chromosome 18. *SMAD4*, located on chromosome 18, a key conveyer of TGFβ signaling and found to be mutated in several types of cancer has been suggested to be haploinsufficient in mouse models [10, 11]. If SMAD4 protein expression is linked to chromosome 18 loss this could provide an example of a mechanism by which CNAs contribute to SINET tumorigenesis in the absence of driver mutations. The overall aim of the present study was to determine if copy number alterations of *SMAD4* and *CDKN1B* in SINETs correlate with corresponding protein expression and can be linked to tumor initiation.

## Methods

### Array comparative genomic hybridization

One hundred thirty-one fresh-frozen SINET biopsies from 117 patients including 52 biopsies from 43 patients previously published [4] were analyzed with array comparative genomic hybridization (CGH). One hundred nine of the 131 CGH analyzed biopsies were also represented on the tissue microarray used for immunohistochemistry and FISH. The purity of tumor biopsies was assessed by light microscopy using hematoxylin and eosin stained sections, and only biopsies with at least 70% tumor cells were analyzed. Genomic DNA was isolated from the biopsies using the DNeasy® Blood and Tissue kit (Qiagen GmbH, Hilden, Germany). The array CGH experiments, including labelling and hybridization of DNA were performed as previously described and according to the manufacturer’s protocol (SureTag DNA Labeling Kit; Agilent Technologies Inc., Palo Alto, CA, USA) [4]. Array CGH analysis was performed using the human genome CGH microarray 4x44K (G4410B/G4426A/G4426B), 2x400K (G4448A), 244 K (G4411B) and CGH-SNP microarray 4x180K (G4890A) (Agilent Technologies Inc.). The probes were annotated against genome build UCSC hg19. Hybridized slides were scanned on an Agilent High-Resolution Microarray Scanner followed by data extraction and normalization using Feature Extraction v.4.0.1.21 (protocol CytoCGH_0209_1x or 2x or 4x_Mar14) (Agilent Technologies). Data analysis was carried out using Agilent CytoGenomics 4.0.3.12 (Agilent Technologies Inc.). The ADM-2 (threshold = 6.0) algorithm was used to identify copy number aberrations across the genome. A log^2^ ratio of ±0.25 and CNAs ≥500 kb was considered as a gain or loss. A log^2^ ratio of > 2 was designated as high-level amplification and a log^2^ ratio of < − 2 was designated as homozygous loss. Chromosome arm gains/losses were determined using a 70% length threshold. The aberrations were checked manually to confirm the accuracy of the calls. Known recurrent germline copy number variations (Agilent female/male CNV reference) were excluded from the analysis.

### Whole exome sequencing

DNA from 9 fresh-frozen tumors and their corresponding normal samples (small intestine, muscle, adipose tissue) were extracted using QIAamp DNA mini kit (Qiagen) and sequencing libraries were prepared using the SureSelect All Exon v3 target enrichment kit (Agilent Technologies, CA). The libraries were sequenced on an Illumina HiSeq 2000 in paired end mode with read length 100 bp. After quality trimming using PRINSEQ [12], the reads were aligned to the human reference genome (hg19) using the BWA-MEM algorithm (http://bio-bwa.sourceforge.net/). Marking of duplicates and base recalibration was performed with Picard (https://broadinstitute.github.io/picard/) and GATK [13], respectively. Single nucleotide variants and small insertions and deletions were called for the *SMAD4* and *CDKN1B* genes using HaplotypeCaller (germline variants) and Mutect2 (somatic variants), as implemented in GATK v4.0.8. Only germline variants with a quality normalized by depth above five, and somatic variants annotated as PASS, were considered. Additionally, variants were required to have a population frequency below 0.10 in the 1000 Genome project.

### Tissue microarray (TMA)

The tissue microarray contained 846 tumor biopsies from 412 patients retrieved from patients who underwent surgery for SINET at Sahlgrenska University Hospital in the years 1986 to 2013. Details of the construction of the tissue microarray have previously been described [14]. The diagnosis of all tumors was re-validated by staining all tumors for hematoxylin and eosin, synaptophysin and chromogranin A, and reviewed by board-certified pathologist (O.N.).

### Immunohistochemistry of TMA and Smad4^+/−^ mice

Immunohistochemistry was performed on SINET TMAs and on gastrointestinal tissue including duodenum, jejunum, ileum from wild-type *Smad4*^*+/+*^ and *Smad4*^+/−^ mice. The *Smad4*^+/−^ mice were derived from F5-F8 backcross generations to C57BL/6 of the original F1 129Ola/C57BL/6Jico SMAD4^+/E6sad^ founder [15] and was kindly donated by Professor Riccardo Fodde. Sections (3–4 μm) from formalin-fixed and paraffin-embedded blocks were placed on glass slides and treated in Dako PT-Link using EnVision™ FLEX Target Retrieval Solution (high pH). The following primary antibodies were used: anti-SMAD4 (B-8, Santa Cruz), anti-p27 (DCS-72.F6, Abcam), anti-chromogranin A (PHE5, Chemicon and EP1030Y, Abcam), anti-synaptophysin (SP11, Abcam), anti-5HT (H209, Dako and LS-B7118, LSBio), and anti-Ki67 (MIB1; Dako). Immunohistochemical staining was performed in a Dako Autostainer Link using EnVision™ FLEX according to the manufacturer’s instructions (DakoCytomation). EnVision™ FLEX+ (LINKER) rabbit or mouse was used for all stainings. Positive and negative controls were included in each run. The fraction of Ki67-positive cells was estimated by manually counting 500–2000 tumor cells per sample, using printouts. For relative quantification of SMAD4 and CDKN1B expression, the total global staining intensity of tumor cells was assessed, meaning localized subcellular i.e. nuclear or cytoplasmatic staining intensity was not individually evaluated. A board-certified pathologist (O.N.) examined the TMA tissue samples using a light microscope with a 4× objective until samples could with a high degree of repeatability be subcategorized as either ‘None’. ‘Low’, or ‘High’.

### Fluorescence in situ hybridization

Fluorescence in situ hybridization (FISH) was performed on 4 μm paraffin sections from the tissue microarray using dual-color fluorescent probes for *SMAD4* and chromosome 18 centromeric region or *CDKN1B* and chromosome 12 centromeric region (FA0590, FA0305, Abnova). Pre-processing of paraffin sections, hybridization to the probe, post-hybridization washing and fluorescence detection were performed according to manufacturer’s instructions (Abnova). Tumors were examined using an Axioplan 2i epifluorescence microscope (Zeiss, Oberkochen, Germany). Within each section, normal regions/stromal elements served as the internal control to assess quality of hybridization. Cases were scored with using a 100× objective lens, counting at least three distinct areas and at least 30 discrete nuclei per area. Labeled nuclei were imaged using an LSM 780 confocal microscope (Zeiss, Oberkochen, Germany). Brightness/contrast adjustments, scale bar, and image type transformation were performed using ZEN 3.1.

### Statistical analysis

Correlation between protein expression and copy number status was estimated with Spearman’s rank correlation coefficient. Comparison of the number of CgA-positive cells in the small intestinal crypts and crypt bases between *Smad4*^+/−^ and *Smad4*^+/+^ mice was performed using unpaired two-sided Student’s t-test. Comparison of SMAD4 expression between tumor sites and patient disease stages was tested with Mann Whitney U-test.

## Results

### Hemizygous loss of chromosome 18 is the most frequent chromosomal aberration in SINETs

**Fig. 1:**
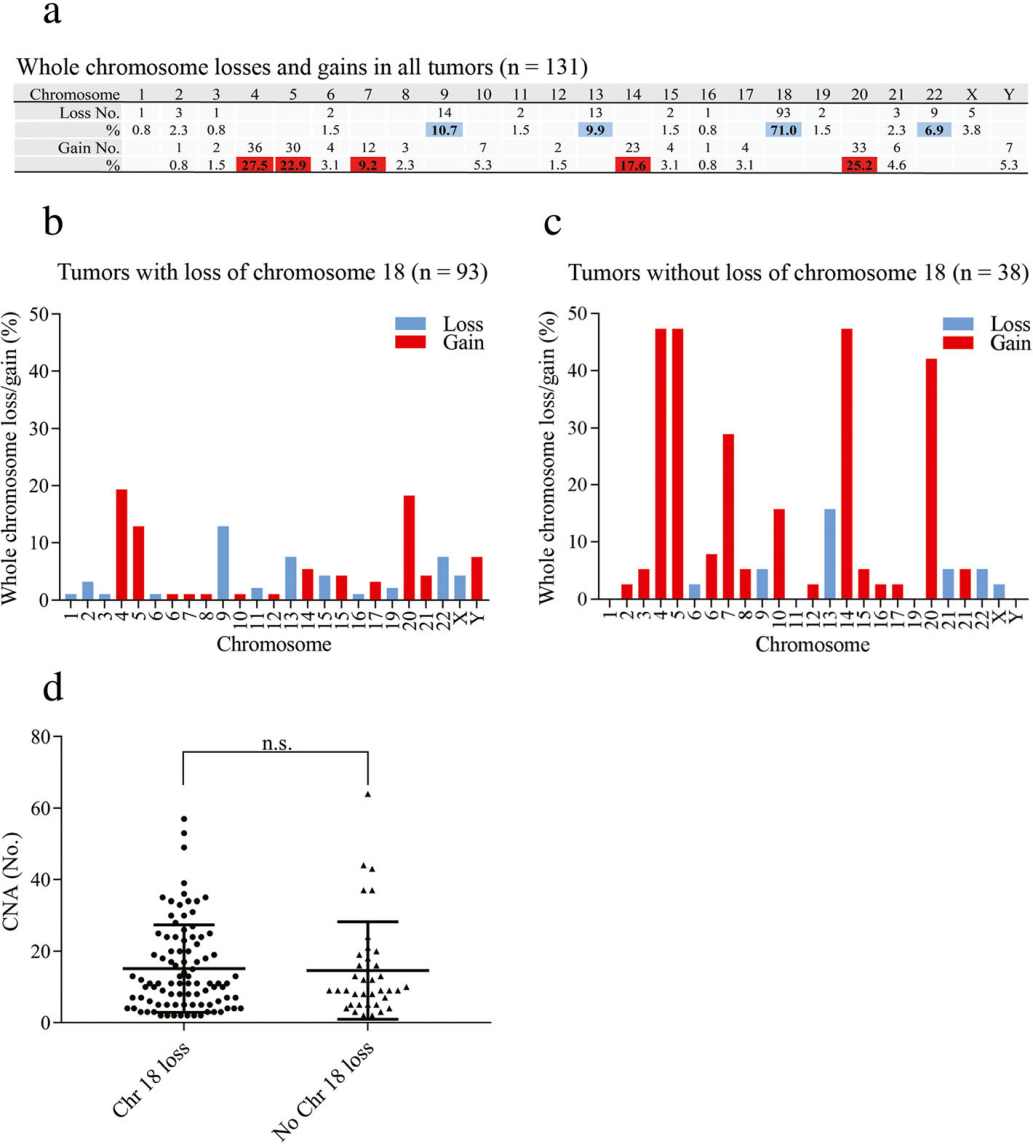
Copy number alterations (CNAs) in small intestinal neuroendocrine tumors (SINETs). **a** CGH analysis of 131 SINETs revealed that the most frequent chromosomal rearrangement is hemizygous loss of chromosomes 9, 13, 18 and 22 and gain of 4, 5, 7, 14 and 20. **b** and **c**. Gain of chromosomes 4, 5, 7, 10, 14 and 20 was more frequent in tumors without hemizygous loss of chromosome 18 (*n* = 38) compared to SINETs with hemizygous loss of chromosome 18 (*n* = 93). (**d**) The average number of CNAs including all fragments larger than 500 kb was similar in tumors with (15.2 ± 1.3) or without loss of chromosome 18 (14.7 ± 2.2)

To investigate and outline recurrent CNAs in SINETs, we performed copy number analysis on 131 SINETs from 117 patients, currently the largest investigated SINET patient cohort. The most frequent CNA was hemizygous loss of chromosome 18 (71%), consistent with previous reports [4, 6] (Fig.1a). Tumors with loss of chromosome 18 and without loss of chromosome 18 differed in the type of harbored chromosomal alterations. Tumors with loss of chromosome 18 had both fewer gains of whole chromosomes as well as more losses of whole chromosomes compared to tumors without loss of chromosome 18 (Fig. 1b, c). Some specific chromosomes, including chromosomes 4, 5, 7, 14, and 20, were more frequently gained in tumors without chromosome 18. CNAs larger than 500 kb were present in all biopsies and there was no significant difference in number of CNAs in SINETs with loss of chromosome 18 (15.2 ± 1.3, *N* = 93) compared to SINETs without loss of chromosome 18 (14.7 ± 2.2, *N* = 38) (Fig. 1d). The CNA analysis did not detect any homozygous deletion of *SMAD4* gene despite the high frequency of hemizygous loss of chromosome 18. *CDKN1B* is located on chromosome 12 and in contrast to chromosome 18, whole chromosome 12 was never lost, but rather harbored focal events. Among the 117 analyzed patients, 11 tumors (9.4%) had loss of the *CDKN1B* gene. The most frequently lost region that included the *CDKN1B* gene was chr12: 11,139,390–12,874,937 which contained 28 genes (Suppl. Fig. 1A–B). In an attempt to identify potential ‘second-hit’ mutations in tumors with loss of *SMAD4* and *CDKN1B* we performed whole exome-sequencing of 9 SINETs and their corresponding normal samples. These 9 tumors were also included in the copy number analysis and 7 tumors had loss of chromosome 18 while none had loss of the *CDKN1B* gene. The sequencing analysis revealed that for these 9 patients no germline or somatic mutations were found neither in the *SMAD4* gene nor the *CDKN1B* gene.

### Hemizygous loss of *SMAD4* leads to lower SMAD4 protein levels

**Fig. 2:**
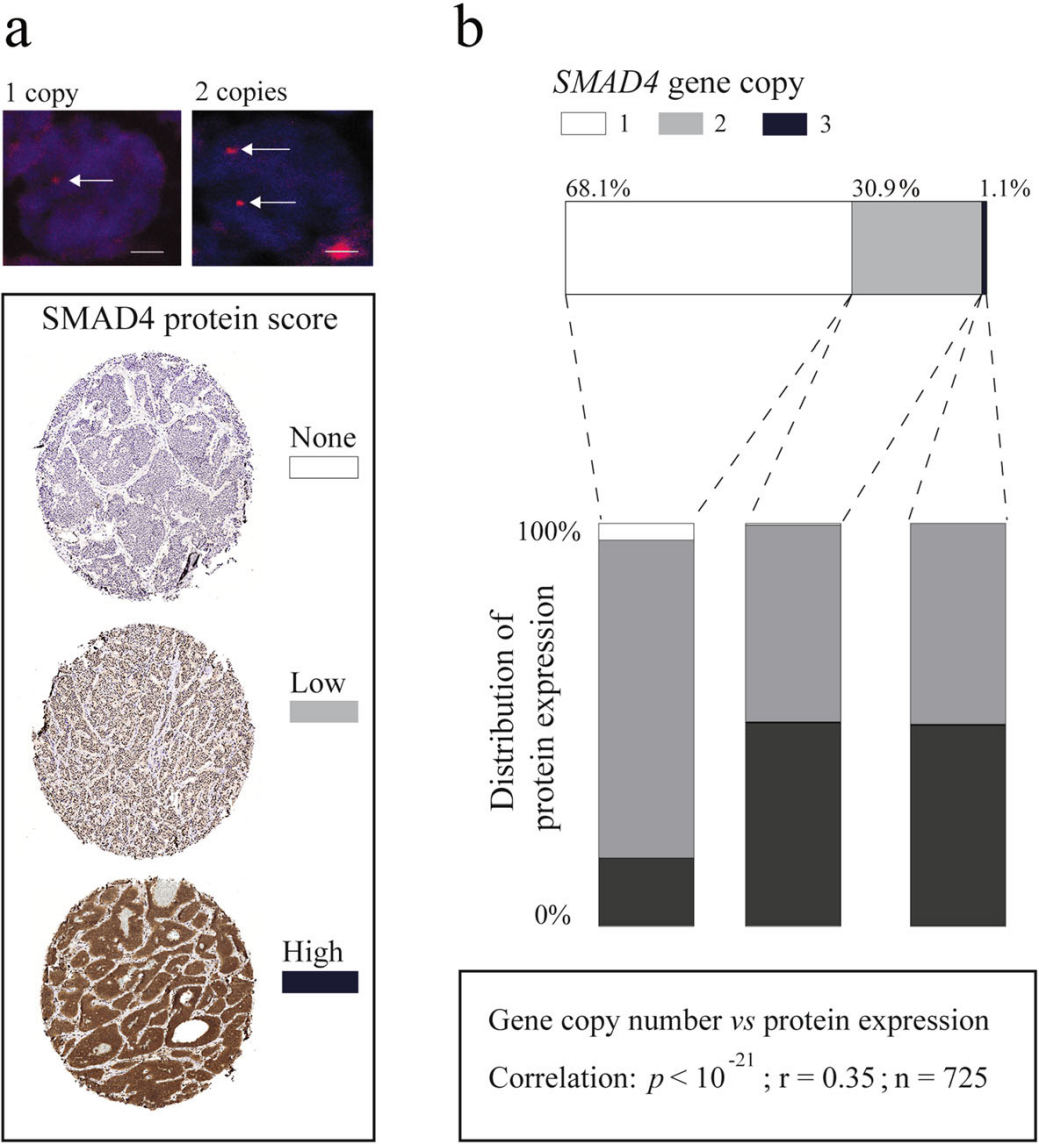
Hemizygous loss of *SMAD4* was associated with lower SMAD4 protein expression. **a** Top: Images visualizing FISH analysis with DAPI stained nuclei (blue) and one respective two Texas red stained copies of the SMAD4 gene (red). Scale bar is equal to 2 μm. Bottom: Images visualizing biopsies from the TMA representing the expression intensities for scoring None, Low or High SMAD4 expression. **b** FISH analysis of a tissue microarray containing 846 tumors from 412 patients showed that hemizygous loss of *SMAD4* (68.1%) occurred recurrently in SINETs. Biopsies on the TMA were both scored for *SMAD4* gene copy number by FISH analysis and for SMAD4 protein expression by immunohistochemical staining. The distribution of SMAD4 protein expression by gene copy number [1–3] is shown. *SMAD4* copy number was positively correlated with SMAD4 protein expression

We investigated the *SMAD4* and *CDKN1B* gene copy number status in 739 and 689 tumors from 373 and 359 patients respectively using fluorescence in situ hybridization (FISH) on tissue microarrays. As expected, loss of *SMAD4* was frequent with 497/739 tumors (68.1%) having one remaining *SMAD4* copy (Fig. 2a). Gains were rare with only 8/739 tumors (1.1%) harboring three *SMAD4* copies. Loss of *CDKN1B* was less frequent compared to *SMAD4* loss, with 45/689 (6.8%) tumors having one copy (Suppl. Fig. 2). *CDKN1B* gains were also infrequent with 13/689 tumors (1.9%) harboring three gene copies. To investigate if *SMAD4* and *CDKN1B* act as haploinsufficient genes in SINETs we assessed whether hemizygous loss leads to decreased amounts of corresponding protein levels. Using immunohistochemistry and FISH, we evaluated the protein staining intensity of SMAD4 and p27 in relation to gene copy number status on consecutive tumor sections. We found that *SMAD4* copy number status had a highly significant correlation to SMAD4 protein staining intensity (Fig. 2b). The number of *CDKN1B* gene copies did however not correlate with the expression of its corresponding protein, p27 (Suppl. Fig. 2).

### Heterozygous inactivation of *Smad4* is not sufficient to induce hyperplasia in enteroendocrine cells

**Fig. 3:**
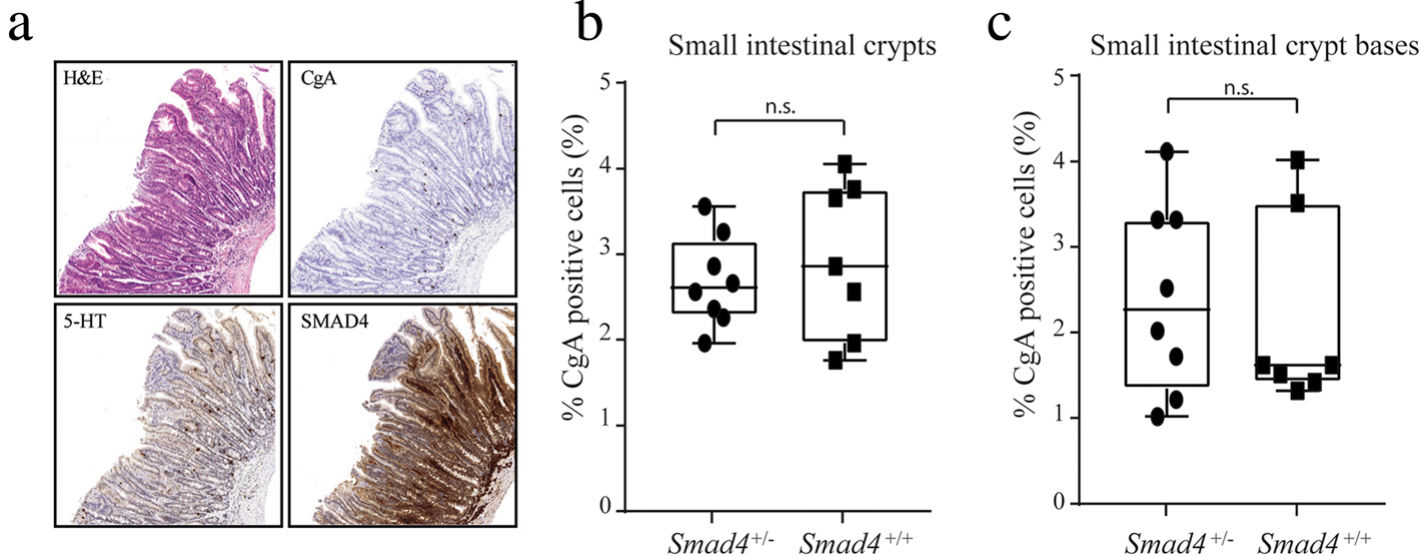
Smad4^+/−^ mice did not develop neuroendocrine tumors or display increased endocrine cell proliferation. **a** One of two low-grade serrated adenomas found in Smad4^+/−^ mice stained with hematoxylin & eosin, anti-chromogranin A (CgA), anti-serotonin (5-HT), and anti-SMAD4. There was no observable difference between number of CgA-positive endocrine cells in small intestinal crypts (**b**) or crypt bases (up to and including position + 4) (**c**)

To investigate if heterozygous *Smad4* inactivation could induce SINET tumorigenesis in mice we used *Smad4*^+/−^ genetic mice. These mice spontaneously develop polyps and adenocarcinomas in the gastrointestinal tract [10, 11, 15]. In our analysis we focused on chromogranin A (CgA)-positive endocrine cells of the small intestine. Paraffin-embedded small intestinal tissue samples from the mice were stained with eosin & hematoxylin, chromogranin A, and serotonin, and were evaluated by board-certified pathologist (O.N.) for presence of tumors. In 2/8 heterozygous mice adenomas with low-grade dysplasia was observed. One sample had a serrated adenoma located in the duodenum and one sample had two early adenomas with low-grade dysplasia (Fig. 3a). No tumors of endocrine origin were observed. In an attempt to identify early stages of hyperplasia, we quantified the number of CgA-positive endocrine cells of the intestinal crypts. We did not observe any differences in the number of CgA-positive cells in the small intestine of *Smad4*^+/−^ and *Smad4* wildtype mice (Fig. 3b). Previous studies have suggested that SINETs originate from CgA-positive cells in the epithelium at the base of the intestinal crypts [16]. We thus also quantified the number of CgA-positive cells located specifically at the bottom of the intestinal crypts (up to and including position + 4), but did again not observe any significant difference between the two genotypes (Fig. 3c). This suggests that heterozygous inactivation of *Smad4* in the mouse intestine is not alone sufficient to sustain or drive endocrine cell proliferation.

### Patients with lower SMAD4 expression in primary tumors more frequently present with metastases

**Fig. 4:**
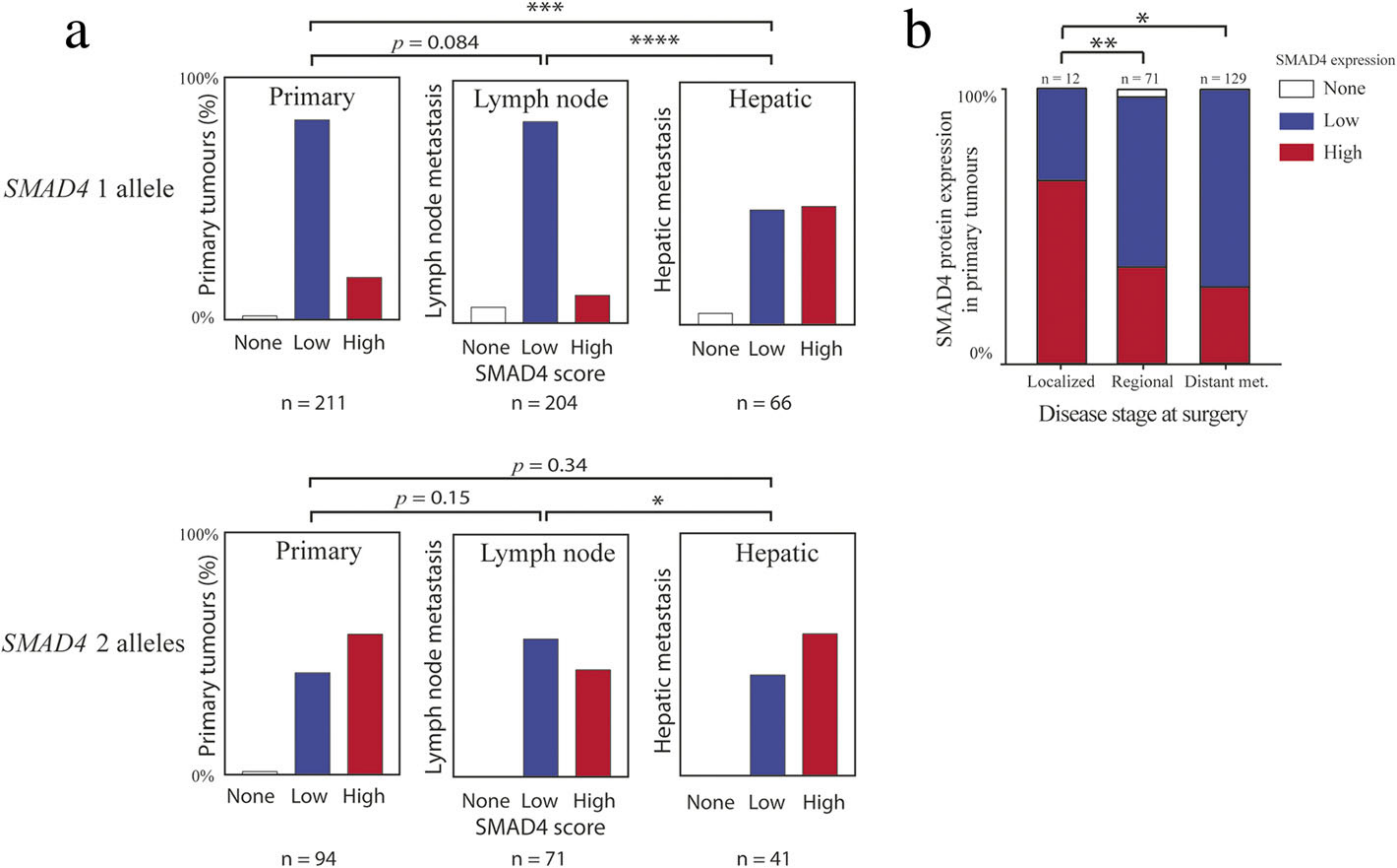
SMAD4 expression varied by tumor site and lower SMAD4 expression in the primary tumor correlated with the patient presenting with metastasis at the time of surgery. **a** Immunohistochemical staining of the SINET tissue microarray revealed that low expression of SMAD4 was more frequent in both primary tumors and lymph node metastases compared to hepatic metastases for tumors with one *SMAD4* copy. **b** Comparing SMAD4 protein expression in the primary tumor between patients with localized disease (stage I, IIA, and IIB), lymph node metastases (stage IIIB), and distant metastases (stage IV) showed that patients with low SMAD4 protein expression in the primary tumors more often presented with metastatic disease. Asterisks indicate statistically significant changes (Mann Whitney U-test): * *p* < 0.05, ** *p* < 0.01, *** *p* < 0.001, **** *p* < 0.0001

The SMAD4 protein expression also differed between tumor sites. In tumors with only one *SMAD4* copy, SMAD4 protein expression was lowest in primary tumors and lymph node metastasis, while hepatic metastases showed a higher SMAD4 protein expression (Fig. 4a). For tumors with two *SMAD4* alleles there was only a significant difference between lymph node metastasis and hepatic metastasis. We also investigated whether SMAD4 protein expression of the primary tumors could predict if the patient presented with distant metastases at the time of surgery. SINET patients are typically staged into disease stages I, IIA, IIB (localized disease with varying tumor invasion), IIIB (tumors with any regional lymph node metastasis), and IV (tumors with any distant metastasis). We found that patients with lower SMAD4 levels in the primary tumor had more often metastasized to regional lymph nodes and distant parts of the body at the time of initial surgery (Fig. 4b). Finally, there was no correlation between Ki67 index and expression of SMAD4 (Suppl. Fig. 3).

## Discussion

Loss and gain of chromosomes, or aneuploidy, is believed to be of importance in tumor development. Although most whole-chromosome or chromosome arm imbalances lead to lower cell fitness when introduced in an experimental setting [17], they are frequently tolerated in cancer cells and about 88% of all cancer types harbor such events [18]. In fact, a solid tumor on average harbors about 3 gains and 5 losses at the chromosomal arm-level [19, 20]. The highly recurrent character of SINET copy number changes suggests that these changes are clonally selected within tumors. Even though aneuploidy in cancer is a common genetic event, due to its complexity, the functional implications are not as well-characterized as specific point mutations. A basic mechanism by which chromosomal alterations could affect the phenotypical characteristics of a cell is through gene dosage, i.e. the number of gene copies influence corresponding protein expression levels.

Initially, we analyzed 131 tumors from 117 patients for chromosomal aberrations confirming hemizygous loss at chromosome 18 as the most common genetic event in our SINET cohort. We also found that losses of chromosomes 9, 13, 18, and 22 and gains of chromosomes 4, 5, 7, 10, 14, and 20 regularly occurred. Chromosome 18 contains the gene encoding the SMAD4 protein that mediates TGFβ signaling and the *SMAD4* gene is frequently mutated in several types of malignant tumors resulting in altered TGFβ signaling and thereby promoting tumor progression and metastatic capacity [21, 22]. We therefore investigated the association between chromosome 18 copies and SMAD4 protein expression in a large SINET cohort. We observed a significant correlation between gene copy numbers and protein expression levels for SMAD4 consistent with a gene dosage mechanism. We also performed a similar analysis of the *CDKN1B* gene located on chromosome 12. As previously mentioned, *CDKN1B* is the most commonly mutated gene in SINETs, albeit at a low frequency. As we observed several segmental losses on chromosome 12 affecting the *CDKN1B* gene we therefor hypothesized that hemizygous loss could result in a lower expression of the p27 protein, suggesting that alterations to *CDKN1B* affected a greater proportion of SINETs and not only those harboring mutations. However, we could not see a similar association between gene copy numbers and protein expression levels for p27 in the SINET samples arguing against a gene dosage mechanism in this context.

Although the functional consequences of reduced SMAD4 protein levels in SINET are unclear, *SMAD4* mutations in other types of cancers shift signaling by TGFβ from growth inhibiting to cancer promoting [21]. Accordingly, based on the lack of TGFβ binding protein (LTBP), Chaudry et al. has speculated that midgut carcinoids are insensitive to TGFβ growth inhibitory effect [23]. For tumor cells to metastasize they need to go through a series of changes, including loss of cell polarity and dependence on cell-cell interactions, termed epithelial to mesenchymal transition (EMT). *SMAD4* inactivation can lead to an accumulation of nuclear-β-catenin resulting in overexpression of β-catenin target genes, promoting EMT and thus metastasis [22, 24]. In this light, our finding that decreased SMAD4 expression in primary tumors was associated with the patient presenting with disseminated disease may indicate that this is an important mechanism also for SINETs. The discrepancy in SMAD4 protein expression between tumor sites could i.e. be explained by differing tissue environment conditions and/or genetic intratumoral heterogeneity between primary and metastatic tumors observed in SINETs [25]. Therefore, further mechanistic studies focused on the effects of disrupted TGFβ-signaling and SINET metastatic capacity could represent a promising future research direction.

Germline mutations in *SMAD4* can lead to juvenile polyposis syndrome (JPS), characterized by benign tumor formation in the gastrointestinal tract and an increased risk of gastrointestinal malignancies. Interestingly, homozygous inactivation is not required for malignant transformation of JPS-related gastro-enteral polyps [26] suggesting gene dosage as a possible mechanism. Among cancer diseases with the highest *SMAD4* alteration frequency are colorectal cancer, pancreatic cancer, esophageal cancer, and gastric cancer, all originating in the GI tract (https://www.cancer.gov/tcga)  [27]. Also for sporadic small intestinal adenocarcinomas, not included in most large pan-cancer data-sets, SMAD4 has been suggested to have a critical role [28].

To determine the effect of heterozygous loss of *SMAD4* on entero-endocrine cell proliferation we quantified intestinal neuroendocrine cells in tissue samples from a genetic mouse model harboring a *Smad4*^+/−^ genotype. We did not detect a higher frequency of enteroendocrine cells in the intestinal crypts or find any sign of enteroendocrine hyperplasia in the *Smad4*^+/−^ mice compared to wild-type mice. We suggest that our findings could indicate that loss of chromosome 18 is not a tumor initiating event but rather represents an early evolutionary advantage during tumor progression. Interestingly, in addition to allelic loss of SMAD4, the genes BCL2, CDH19, DCC have also been reported as potential candidate haploinsufficient genes and gains of oncogene SRC located on chromosome 20 has previously been found to correlate with a poorer prognosis [3]. Thus, further explorations regarding the effects of haploinsufficiency and SINET could provide important insights regarding the SINET tumor biology.

## Conclusions

In summary, *SMAD4* copy number correlated with corresponding protein expression in a haploinsufficient manner. The findings in this study suggest that copy number alterations in SINET can affect protein expression of known oncogenes and CNA induced gene-dosage could represent a novel mechanism regarding SINET biology. Further research regarding functional implications of reduced SMAD4 expression in SINETs is warranted.

## Supplementary Information

**Additional file 1.**

**Additional file 2.**

**Additional file 3.**

## Data Availability

The datasets are available according to the MIAME standard at the Gene Express Omnibus database (https://www.ncbi.nlm.nih.gov/geo/) with accession number GSE153314.

## Abbreviations

CDKN1B: Cyclin-dependent kinase inhibitor
CNA: Hemizygous copy number alteration
EC: Enterochromaffin
FISH: Fluorescence in situ hybridization
PTEN: Phosphatase and tensin homolog
SINETs: Small intestinal neuroendocrine tumors
SMAD4: SMAD family member 4
TMA: Tissue microarray
