# Supplementary figures and images for: SMAD4 haploinsufficiency in small intestinal neuroendocrine tumors
Source: BMC Cancer. 2021 Jan 28;21:101. doi: 10.1186/s12885-021-07786-9 (PMC7841913; doi:10.1186/s12885-021-07786-9)

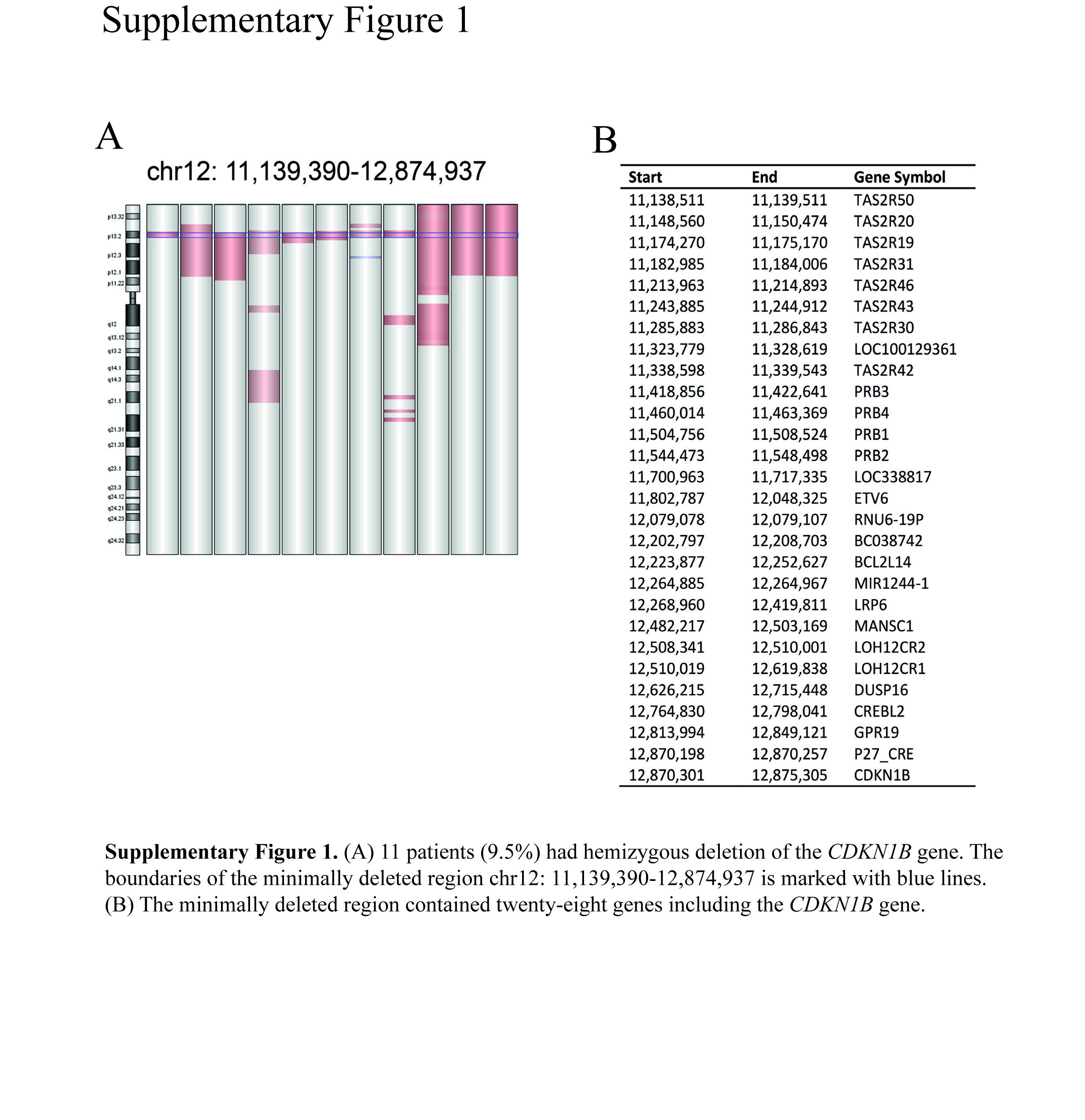

Supplement: Supplementary file 1 — Additional file 1. [file 12885_2021_7786_MOESM1_ESM.tif]

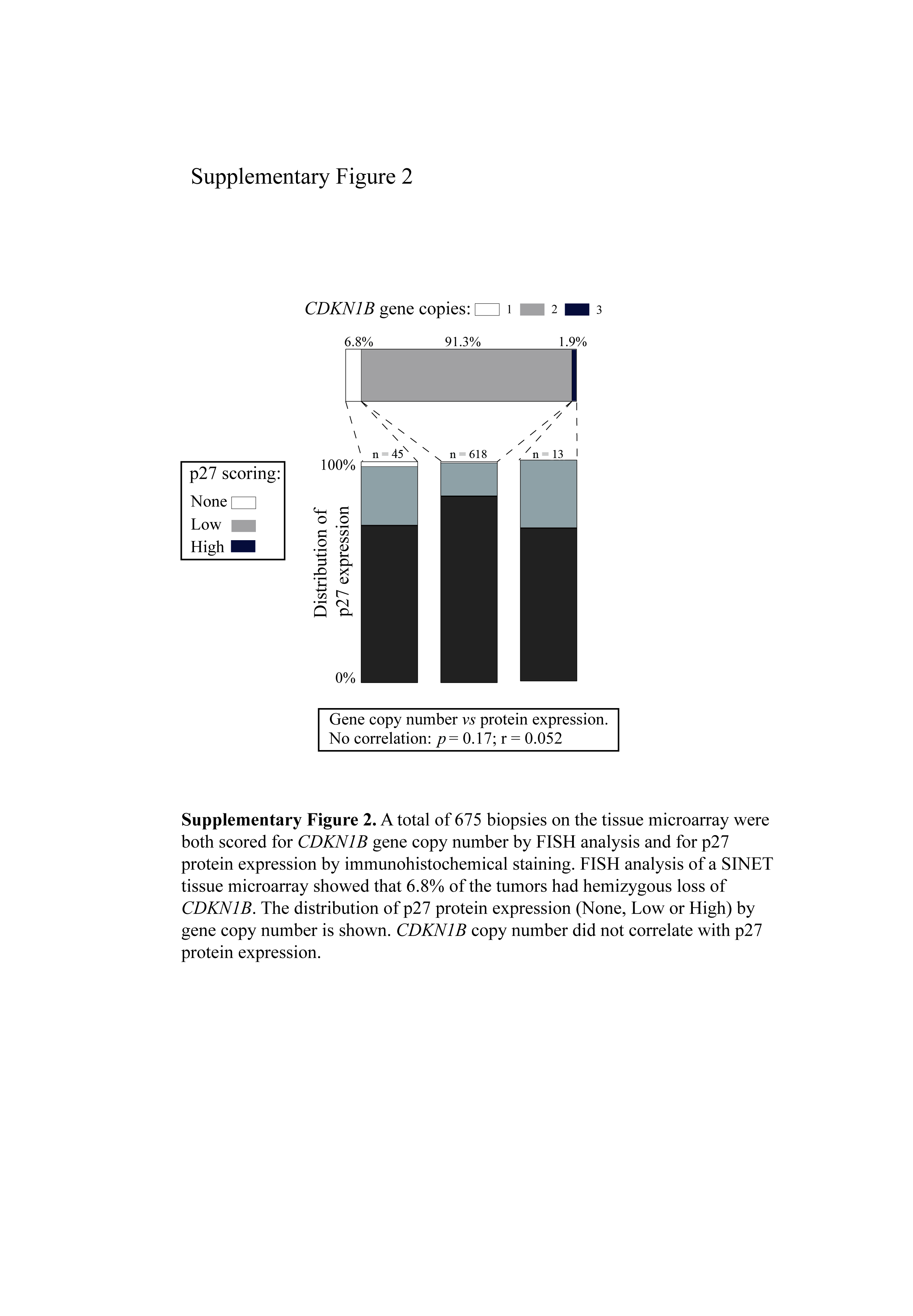

Supplement: Supplementary file 2 — Additional file 2. [file 12885_2021_7786_MOESM2_ESM.tif]
