# Supplementary material for: SMAD4 haploinsufficiency in small intestinal neuroendocrine tumors
Source: BMC Cancer. 2021 Jan 28;21:101. doi: 10.1186/s12885-021-07786-9 (PMC7841913; doi:10.1186/s12885-021-07786-9)
Supplement: Supplementary file 3 — Additional file 3. [file 12885_2021_7786_MOESM3_ESM.pdf]

Supplementary Figure 3

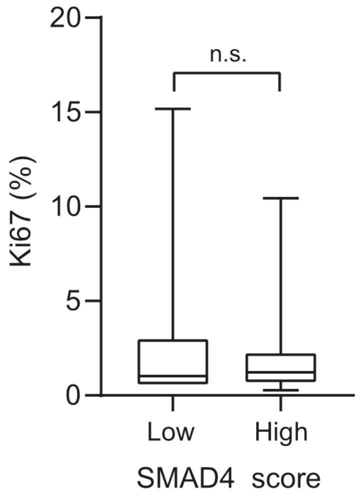

Supplementary Figure 3. The Ki67 index of sixty-four formalin-fixed paraffin-embedded SINETs was determined by board-certified pathologist. There was no significant difference in Ki67 score comparing tumors with a low or high expression of SMAD4.
